# Long-Term Variability in the Content of Some Metals and Metalloids in *Aesculus* Flowers: A Four-Year Study Using ICP OES and PCA Analysis

**DOI:** 10.3390/molecules30040908

**Published:** 2025-02-15

**Authors:** Veronica D’Eusanio, Elia Frignani, Andrea Marchetti, Laura Pigani, Mirco Rivi, Fabrizio Roncaglia

**Affiliations:** 1 Department of Chemical and Geological Sciences, University of Modena and Reggio Emilia, 41125 Modena, Italy; elia.frignani@unimore.it (E.F.); andrea.marchetti@unimore.it (A.M.); laura.pigani@unimore.it (L.P.); mirco.rivi@unimore.it (M.R.); fabrizio.roncaglia@unimore.it (F.R.); 2 National Interuniversity Consortium of Materials Science and Technology (INSTM), 50121 Firenze, Italy; 3 Interdepartmental Research Center BIOGEST-SITEIA, University of Modena and Reggio Emilia, 42124 Reggio Emilia, Italy

**Keywords:** horse-chestnut, chemical composition, elemental analysis, ICP OES, PCA

## Abstract

This study investigates the content of some metals and metalloids in the flowers of three *Aesculus* cultivars (AHP, *Aesculus hippocastanum* pure species, with white flowers; AHH, *Aesculus hippocastanum* hybrid species, with pink flowers; and AXC, *Aesculus × carnea*, with red flowers) over a four-year period (2016–2019) using inductively coupled plasma optical emission spectrometry (ICP OES) and principal component analysis (PCA). The research focuses on assessing macro- and micro-elemental compositions, identifying variations in mineral uptake, and exploring potential correlations with soil composition. Results highlight significant differences in elemental profiles among the three species, despite similar total ash content. Potassium and phosphorus emerged as dominant macroelements, with AXC showing lower magnesium levels compared to AHP and AHH. Particularly intriguing was the detection of antimony in all cultivars, raising questions about its role and bioaccumulation pathways in floral tissues. Iron and aluminum concentrations varied significantly across species, indicating species-specific metal transport mechanisms. Nickel content showed temporal fluctuations, potentially influenced by climatic conditions and soil properties. PCA revealed distinct clustering patterns, linking elemental concentrations to specific species and years. This comprehensive analysis enhances understanding of metal absorption and distribution in ornamental plants, providing insights into their metabolic processes and potential implications for environmental monitoring and phytoremediation strategies.

## 1. Introduction

The plant kingdom represents the foundational link in the terrestrial food chain, making agro-vegetal primary production the principal pathway for transferring metals and mineral substances to the entire animal kingdom [1]. Understanding the composition of the mineral fraction in plants is critical for various scientific and practical objectives, including agriculture, pharmacology, and environmental sustainability [2,3]. It is well-documented that the absorption of mineral constituents in plants is governed by intrinsic physiological mechanisms [4], significantly influenced by the geochemical characteristics of the soil and the selective uptake capacity of plant species [5]. These species exhibit variability in accumulating specific elements across different plant tissues, such as roots, bark, stems, flowers, fruits, and leaves [6,7]. Generally, plants tend to sequester potentially toxic elements in vegetative organs like roots, stems, and leaves, while limiting their accumulation in reproductive organs such as flowers, fruits, and seeds, as a survival strategy to safeguard reproduction and species propagation. However, flowers can serve as indicators of elemental transport and partitioning within plants, offering unique insights into physiological mechanisms of uptake, translocation, and distribution. The flowers of *Aesculus* species, in particular, have additional relevance due to their known medicinal importance and potential interactions with pollinators such as bees. These plants are melliferous [8], and any toxic element accumulation in flowers could impact pollinator health and honey production, further highlighting the ecological importance of studying flowers in this context [9,10]. While their limited availability during the growth season poses sampling challenges, flowers represent a valuable temporal snapshot of elemental distribution in reproductive tissues, complementing data from other plant organs. Furthermore, mineral absorption processes are sensitive to secondary effects caused by anthropogenic impacts, such as pollution and soil degradation, which can disrupt natural uptake and distribution mechanisms. These disruptions highlight the importance of studying plant mineral composition across various environmental contexts and time periods [11].

Despite significant research efforts, particularly in recent decades, many questions remain unresolved regarding plant mineral composition and physiology, particularly for ornamental species with pharmacological potential. Within this context, our research group has focused on *Aesculus* species cultivated on the university campus of Modena, Italy, with particular attention to their seeds [12,13,14,15]. These ornamental plants belong to the *Sapindaceae* family and include at least three distinct types: AHP (*Aesculus hippocastanum* pure species with white flowers), AHH (a hybrid species identified by botanists, characterized by pink flowers), and AXC (*Aesculus × carnea*, with red flowers). This diversity provides an excellent framework for exploring the variations in composition among closely related plant types and understanding how these variations are influenced by environmental factors.

Over the past 20–30 years, there has been a growing scientific interest in the pharmacological and cosmeceutical potential of biomolecules derived from the *Hippocastanoideae* subfamily [16]. This surge in research can be attributed to the promising therapeutic applications of these compounds, particularly those found in the seeds. The seeds, rich in triterpenic saponins such as escin, are well-known for their potent anti-inflammatory properties and have been extensively studied in pharmacological contexts [17,18]. However, other vegetative parts, including bark, leaves, and flowers, remain underexplored, resulting in significant knowledge gaps [19,20,21,22]. Recent studies have highlighted the presence of biologically active compounds in the flowers of the *Hippocastanoideae* subfamily, particularly *Aesculus hippocastanum* (AH), including flavonoids, coumarins, carotenoids, polyphenolic compounds, and essential oils [19,23]. Dudek-Makuch et al. [19,20] reported flavonoid and coumarin compounds in AH flowers, which are recognized for their health benefits and therapeutic properties. Similarly, Deli et al. [22] identified variations in carotenoid composition within AH and *Aesculus pavia* flowers, correlating these differences to the chromogenic properties of the flowers’ distinctive streaks. Further, Owczarek et al. [23] detected approximately 40 polyphenolic compounds in horse chestnut flower using advanced UHPLC techniques, indicating their diverse bioactive potential. Essential oils derived from fresh AH flowers, as characterized by Bobaev et al. [24] using GC-MS, exhibited notable antibacterial and antifungal properties attributed to specific bioactive molecules [25]. Despite these findings, prior studies have primarily focused on the chemical and biological activity of these flowers, leaving other critical aspects, such as their mineral composition, unexplored. This represents a notable gap in understanding the broader pharmacological and ecological significance of these plant materials.

Addressing these gaps, this study aims to provide foundational insights into the mineral composition of AH flowers, an area that has received scant attention in the literature. The research focuses on identifying, monitoring, and quantifying selected metals in the flowers of three distinct *Aesculus* species, AHP, AHH, and AXC, cultivated on the university campus in Modena, Italy. Conducted over four consecutive years (2016–2019), the study employs a principal component analysis (PCA) to uncover patterns and correlations within the data. Additionally, the mineral content of soil samples collected in 2016 and 2019 was analyzed to examine potential relationships between soil composition and mineral uptake in the flowers. By providing a long-term, multi-species dataset, this study addresses the limitations of prior research, such as its narrow focus on bioactive compounds and its geographic constraints. Moreover, the findings have practical implications for pharmacological research, environmental monitoring, and sustainable management of ornamental plants. By addressing the inherent limitations of prior studies, such as their narrow focus on specific bioactive compounds or geographic constraints, this study seeks to expand the existing database [15] and contribute to a deeper understanding of the mineral profiles of these plant materials. This comprehensive approach underscores the significance and necessity of exploring understudied aspects of AH flowers, thereby advancing both scientific knowledge and potential applications.

## 2. Results and Discussion

### 2.1. Proximate Analysis

Proximate analysis serves as a critical first step in the comprehensive characterization of plant raw materials [26,27,28]. This analytical approach establishes a foundation for understanding the nutritional, functional, and biochemical properties of the material under investigation.

Table 1 provides results from a proximate analysis of the flowers from the three examined *Aesculus* genotypes, collected during the four-year study period.

A comparative analysis of the moisture content in fresh flowers reveals that the average values over four years of observations follow the sequence:

Moisture content (% in fresh flowers): AHP (79.9%) > AHH (78.0%) > AXC (77.7%).

The AHP species exhibits a notable difference of approximately 2 percentage points compared to the other genotypes, AHH and AXC. This elevated moisture content in AHP flowers may be closely associated with their higher ash content. A potential explanation for this phenomenon could be linked to the relationship between salinity in the growth environment and water retention capacity. Specifically, higher salinity may promote greater water retention for two key reasons:

(i). To sustain vital physiological processes in an environment governed by osmotic balances, particularly those involving inorganic salts;
(ii). To maintain sufficient quantities of solvation and crystallization water for inorganic and metal–organic compounds without compromising the availability of solvent required for lymphatic transport processes.

Table 2 presents comparative data highlighting the trends in mineral ash content between the seeds [15] and flowers of the three investigated cultivars.

The initial, and somewhat unexpected, experimental finding reveals that the mineral content in flowers consistently surpasses the bulk undifferentiated content found in seeds. On average, the mineral fraction in flowers is approximately 122% higher in the pure species AHP, 142% higher in the hybrid genus AHH, and 66% higher in the AXC species. Furthermore, an analysis of the average mineral fraction in flowers and seeds, conducted over the four years of observations, highlights the following trends:

- ash content in flowers (dry basis):
  AHP (5.50%) > AHH (5.22%) > AXC (5.16%);
- ash content in seeds (dry basis):
  AXC (3.10%) > AHP (2.47%) > AHH (2.25%).

These results underscore a consistent pattern of higher mineral accumulation in the flowers compared to the seeds across all species.

This experimental finding poses a challenge to rationalize within the framework of our current limited knowledge. Particularly intriguing is the observed inverse proportionality between the mineral content in flowers and seeds; as the concentration of the inorganic fraction increases in the flowers, it correspondingly decreases in the seeds, and vice versa.

The mineral fraction, commonly referred to as “ashes,” derived from these plant systems is predominantly composed of metal oxides (M_x_O_y_), phosphate salts, and carbonate salts, with minor contributions from chloride and sulfate salts. The presence of basic metal oxides in the ashes is evidenced by their alkaline pH values (8.5 ≤ pH ≤ 9.5) when dissolved in water. This observation aligns with the understanding that plant roots primarily absorb metal hydroxides [M^+n^(OH)_n_] from physiological soil solutions. Once absorbed, these hydroxides likely undergo salification and complexation with acidic organic compounds and other metabolites produced through basal metabolic processes.

These metal–organic complexes are subsequently integrated into the plant’s dry matter and fully degrade during incineration at 550 °C, releasing the metals in the form of oxides. Together, these findings contribute to understanding the mechanisms of mineral absorption, distribution, and transformation within plant tissues, highlighting the dynamic interplay between flowers and seeds in regulating mineral content.

**Figure 1:**
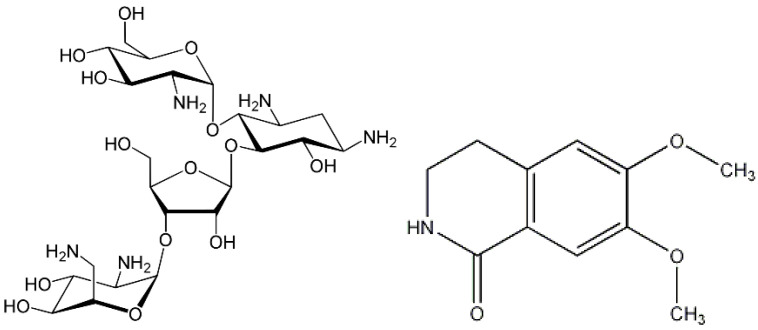
Chemical structures of some compounds present in essential oils extracted from flowers of *Aesculus* species; paromomycin (**left**) and corydaldine (**right**).

The protein content (%) of the *Hippocastanaceae* and *Aesculus* flowers studied in this work shows minimal variation over the four years of monitoring. The average values follow this sequence: (12.9 ± 0.7) % for AHH, (12.6 ± 0.6) % for AXC, and (12.5 ± 0.8) % for AHP. The differences in the absolute values are minimal, well within comparable ranges, and align closely when considering their associated dispersion through standard deviations. Given the indirect method used to measure protein content, it is important to note that AH flowers also contain other non-protein nitrogenous compounds, as evidenced by recent literature [29]. To account for these, we applied a reduced conversion factor of 5.85 [30] for correction and compensation. Among these non-protein compounds, notable examples include paromomycin (Figure 1, left), an aminoglycoside antibiotic derived from *Streptomyces rimosus* var. paromomycinus, known for its amoebicidal and antibacterial properties; corydaldine (Figure 1, right), a natural anti-inflammatory; and carboxamido compounds with antimicrobial effects, all of which are naturally present in the essential oil extracted from AH flowers.

### 2.2. ICP OES Determinations

Table 3, Table 4 and Table 5 present the results related to the metal content in the flowers of the three *Aesculus* species investigated: AHP, AHH, and AXC, respectively. The values in the tables are organized into two groups: (i) major elements, with concentration expressed on a mg/100 g dry weight (d.b.) scale; (ii) minor elements and trace elements, with concentration expressed on a μg/100 g (d.b.) scale.

The statistical analysis performed using ANOVA and the Tukey–Kramer test revealed that, for the majority of the tested elements, there were no significant differences (*p* > 0.05) in mean values across the four years of study. This consistency suggests that the mineral composition of the *Aesculus* flowers is relatively stable over time, despite potential interannual variations in environmental conditions. Such stability could reflect the inherent physiological mechanisms of these species, which might buffer against minor fluctuations in soil composition or external factors. However, a few significant differences (*p* < 0.05) were observed for certain elements, which can likely be attributed to variations in meteorological conditions, such as changes in rainfall, temperature, or seasonal patterns, during the years of study. Meteorological fluctuations are known to influence soil chemistry, nutrient availability, and plant metabolism, thereby affecting the uptake and accumulation of specific minerals in plant tissues. For instance, increased rainfall in certain years may have led to nutrient leaching in the soil, reducing the bioavailability of some elements, while drier conditions could have concentrated nutrients in the upper soil layers, making them more accessible to plant roots. Variations in temperature and humidity may alter the transpiration rates of plants, which, in turn, can affect nutrient transport and accumulation within different tissues. Additionally, changes in sunlight exposure or seasonal shifts might have influenced the phenological development of the flowers, leading to differences in mineral composition.

**Figure 2:**
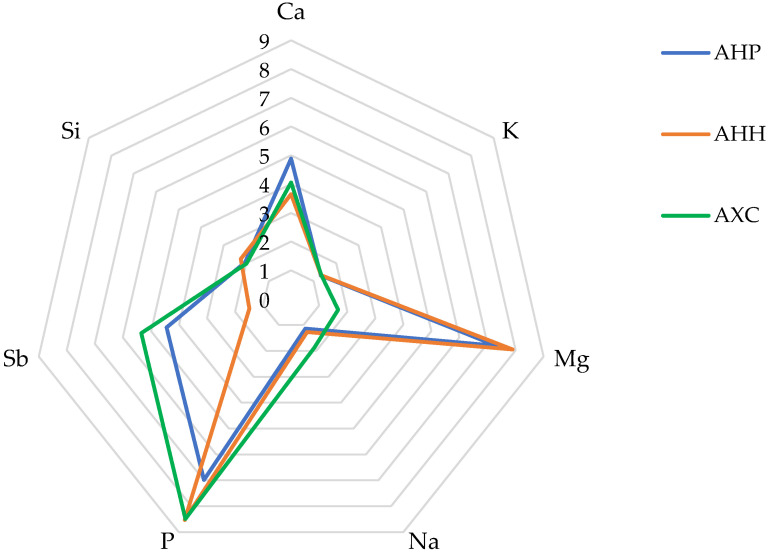
Radar plot depicting the concentration of major elements determined in AHP, AHH and AXC flower samples. The data have been scaled to better visualize the differences between samples (Ca: mg/100 g × 10^−1^; K: mg/100 g × 10^−3^; Mg: mg/100 g × 10^−1^; Na: mg/100 g × 10^−1^; P: mg/100 g × 10^−2^; Si: mg/100 g × 10^−1^; Sb: mg/100 g).

To facilitate comparison, the average concentration values of the major analytes are presented in Figure 2 in the form of a radar plot, with appropriate scaling.

The element distribution profiles shown in Figure 2 highlight the tightly clustered concentrations of two analytes, K and Si, in the flowers of all three cultivars. Following these, progressively larger variations are observed for Na, Ca, and P. In contrast, Mg shows relatively consistent values in AHP and AHH, while AXC displays a significant deviation, with its average concentration reduced to approximately one-fifth of the levels found in the other two cultivars.

Potassium is well recognized as the most abundant metal in plant systems where, along with Na, it plays a critical role in the primary lymphatic-cellular osmoregulatory system, in addition to other regulatory functions. For the three *Aesculus* varieties, the average K content in flower samples ranges from 1.316% (d.b.) in AHP to 1.339% (d.b.) in AXC.

Table 6 reports the average values over four years of study (2016–2019) of the content of the three fundamental elements for the life of plant systems (N, P and K), and the related ratios.

As shown in Table 6, the AHH species has the highest average N % content, slightly exceeding the AHP and AXC varieties, which both stand at approximately 2.15%. Conversely, the P % content is nearly identical between the AHH and AXC varieties, while the AHP species exhibits a significantly lower value, approximately 18% less. This observation suggests that the AHP species may have reduced phosphorus requirements compared to the AHH hybrid and AXC. The K % content appears relatively stable across all three cultivars. However, the lower P % content in the AHP species results in a particularly favorable N %/P % ratio, exceeding the values of the other two cultivars by approximately 17%. In contrast, the N %/K % ratio remains consistent across the varieties. Finally, the P %/K % ratio highlights a different trend, with the AHH and AXC cultivars surpassing the AHP species by approximately 18%. These findings may reflect differences in nutrient uptake efficiency or physiological needs among the species [31,32,33,34]. For instance, the lower P % requirement of the AHP species could indicate an adaptation to phosphorus-deficient soils, making it a promising candidate for cultivation in environments where phosphorus availability is limited. Conversely, the higher P %/K % ratio observed in the AHH and AXC suggests these cultivars may prioritize processes like energy metabolism and cellular function, which are heavily reliant on phosphorus availability [35]. The stable K % content across all species highlights its fundamental role in maintaining essential physiological functions, such as osmoregulation and enzyme activation [36]. However, the observed variations in N %, P %, and their respective ratios underscore the potential influence of genetic traits or environmental factors on nutrient dynamics.

Silicon, another element present in similar concentrations across all three cultivars, averages approximately 0.02% (d.b.). While the role of Si in plants is not yet fully understood [37], studies such as those by Strout et al. [38] suggest that silica (SiO_2_) significantly influences the structural coloration of leaves, which has substantial implications for the quantum efficiency of photosynthesis. Likewise, silica nanoparticles are thought to play an important role in determining flower coloration, aiding in the attraction of pollinating insects through visual cues. Silica absorbed through the root system becomes bioavailable to the lymphatic circuit in the active form of soluble silicic acid (H_4_SiO_4_ · nH_2_O). Under suitable conditions, this compound can polymerize into amorphous hydrated silica [(SiO_2_)n · mH_2_O]. This amorphous polymer, being highly insoluble, precipitates as micro- and nano-sized phytolithic micelles within various plant tissues. This biomineralization process plays a crucial role in reinforcing plant tissues, enhancing structural rigidity and increasing resistance to environmental stresses. Floral organs typically exhibit shorter lifespans compared to other plant structures, such as leaves and fruits. Consequently, plants may have evolved mechanisms to utilize amorphous silica, the primary compound of silicon, for the formation of phytoliths that provide structural support to the sepals and petals of the floral calyx. Experimental studies indicated that elevated silicon concentrations improve plant resistance to a wide range of biotic stresses, including those caused by fungi, bacteria, and viruses. Furthermore, silicon plays a crucial role in mitigating abiotic stresses caused by extreme environmental conditions, such as water scarcity and oxidative stress, which result from environmental imbalances. These hypotheses, strongly supported by evolutionary biologists and corroborated by experimental evidence, suggest that plants strategically employ silicon to enhance their defense systems and minimize the adverse effects of external stressors. This adaptive advantage is particularly critical for pollination, a process essential for plant reproductive success [39]. In addition, the active form of silicic acid may contribute to the synthesis of organic defense compounds against a variety of pathogens by modulating gene expression. Despite these insights, significant gaps remain in understanding the pathways of silicon transfer within plants, the mechanisms underlying both active and passive accumulation in vegetative tissues involved in phytolith formation, and the specific genes encoding silicon transporters [40].

Table 3, Table 4 and Table 5, and Figure 2 reveal an unexpected finding in light of current knowledge: the presence of Sb among the major elements. The average concentrations of Sb are lowest in AHH (1.49 mg/100 g d.b.), intermediate in AHP (4.44 mg/100 g d.b.), and highest in AXC (5.34 mg/100 g d.b.). While Sb is the least abundant of the seven major elements detected, its concentrations consistently exceed the classification threshold of 1 mg/100 g d.b. Antimony is a non-essential trace element for both primates and humans, with documented toxic effects, particularly among individuals exposed through occupational activities. Symptoms of Sb poisoning are similar to those caused by As. The World Health Organization (WHO), the International Agency for Research on Cancer (IARC) and the European Food Safety Authority (EFSA) classify Sb as a potentially carcinogenic agent [41,42]. The recommended maximum exposure limit is approximately 6 µg/kg of body weight, equivalent to ~360–400 µg per person. The elevated levels of Sb in the flower samples analyzed raise several important questions:

- What role, if any, does Sb play in plant physiology?
- Does its prevalence in *Aesculus* flowers suggest a tendency for bioaccumulation (B-Sb) in storage systems?
- Are there variations in B-Sb concentrations among different flower components in *Aesculus* species?
- How does the concentration of B-Sb in flowers correlate with its levels in other plant tissues?
- Could specific proteins or transport mechanisms in *Aesculus* facilitate Sb transport or storage?

**Figure 3:**
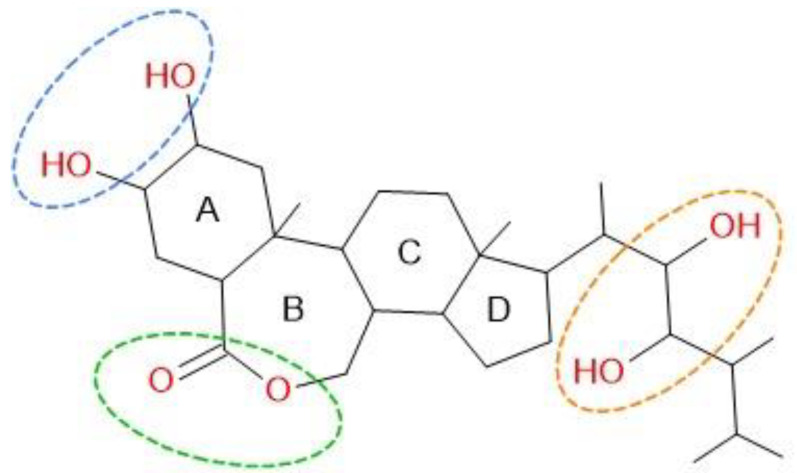
Substituent groups responsible for forming coordination compounds in brassinosteroids (BRs).

Definitive answers to these questions remain unavailable. However, the existing literature offers some insights into potential explanations for these observations. Antimony exhibits bioactive properties primarily in its +3 oxidation state, although the +5 state is also found in modest concentrations within storage systems. These properties parallel those of arsenic, as both elements share a position in the same group of the Periodic Table and exhibit medium amphoteric behavior, with slight differences in chemical characteristics. At the rhizosphere level, the principal species crossing root membranes are Sb(OH)_3_ and As(OH)_3_, while pentavalent species Sb(V) and As(V) are transported to a much lesser extent. No essential function for Sb or As has been identified in plants, regardless of the analytical form. However, an in vitro study using *Escherichia coli* demonstrated that both As(III) and Sb(III) are absorbed from the culture medium through the glyceroporin-facilitator (GlpF) channel, a member of the aquaporin (AQP) superfamily [43,44]. This membrane protein superfamily was first identified in 1999, and since then, AQPs have been found in all domains of life, including bacteria to yeast, plants and human [45]. In neutral solutions, As(OH)_3_ forms a hydroxy acid with three hydroxyl (-OH) groups, exhibiting a moderately weak acidic character. In physiological solutions, whether in plant or animal systems, the pH is generally buffered. This buffering effect may prevent even the dissociation of the first proton, effectively keeping As(OH)_3_ in its neutral molecular form. Under these conditions, the molecule exhibits polarity similar to that of glycerol. Aquaglyceroporins, which regulate the uptake and exchange of glycerol and water, appear to accommodate this structural mimicry, facilitating the absorption of As(OH)_3_ and Sb(OH)_3_. This suggests a shared pathway for the uptake of these metals/metalloids in plants [46]. Tisarum et al. [47] observed that Sb(III) is readily accumulated in the roots of *Pteris vittata* L. However, unlike As, Sb(III) is not extensively transported to the shoots of this species, which is an exceptional As hyperaccumulator. In contrast, *Pteris cretica* L., another species in the *Pteridaceae* family, demonstrates comparable accumulation values for As and Sb in its transport systems [48]. Quantitatively, the transfer processes for pentavalent arsenate (As(V)) and antimoniate (Sb(V)) are significantly less efficient than those for trivalent forms As(III) and Sb(III). However, distinct transport systems seem to mediate the movement of Sb(V) and As(V), likely in their anionic forms H_2_MeO_4_^−^, HMeO_4_^2−^, and, to a lesser extent, MeO_4_^3−^ [49]. Unlike As(V), which can substitute for the phosphate ion and exploit phosphate transport systems, Sb(V) does not appear to utilize these mechanisms. The processes governing Sb(V) transport remain poorly understood, requiring further research to clarify these mechanisms This is particularly critical as Sb(V) is recognized as the most toxic form of antimony for plant systems [50]. Further insights are provided by Sharma et al. [51], who discussed the role of brassinosteroids (BRs), a class of phytohormones belonging to the steroid hormone family, in regulating plant responses to metalloids such as As and Sb, along with Se and other elements. The molecular structure depicted in Figure 3 illustrates the substituent groups responsible for forming coordination compounds, both with free or partially solvated metal cations (notably through electron-donating oxygen atoms of ester groups) and via hydrogen-bond bridges (H-bridges) with molecular analytes such as Me(OH)_n_. The active -OH groups in BRs can act as either hydrogen bond donors (DHB) or acceptors (AHB), providing a versatile framework for interaction. These features suggest that BRs may be highly effective, albeit poorly selective, in transporting various metallic and metalloid species within vegetative tissues.

Moreover, the functional versatility of BRs extends to multiple regulatory roles in fundamental biological processes. These include modulating signal transduction pathways, primary and secondary metabolic activities, biomolecular crosstalk, redox homeostasis, and more. However, despite their regulatory potential, BRs do not appear to sufficiently restrict the absorption of metalloids from the environment. Consequently, high concentrations of metalloids in soils can lead to their excessive accumulation in plant tissues, resulting in phytotoxicity and biomagnification within plants. It is well-documented that physiological solutions circulating in soils transport hydrated ions or cations in various forms: partially hydroxylated species [Me^n+^(OH)_m<n_]^∆+^, fully hydroxylated species [Me^n+^(OH)_n_], or complexed hexahydrate ions [Me(H_2_O)_6_]^n+^. These ions must traverse the cellular barriers of plant roots to access the vegetative systems via specialized transport channels. Apart from alkali metals, which exhibit unique chemical behavior (e.g., a single oxidation state of +1 and weak coordination affinity except with oxygen), the metallic species present in plants are generally part of complex molecular systems. In these systems, cations often serve as coordination centers for transport molecules containing active sites composed of heteroatoms capable of forming bonds. Examples include free acids, amino acids, carboxylic group carriers, tannic compounds, polyphenols, enzymes, and proteins.

**Figure 4:**
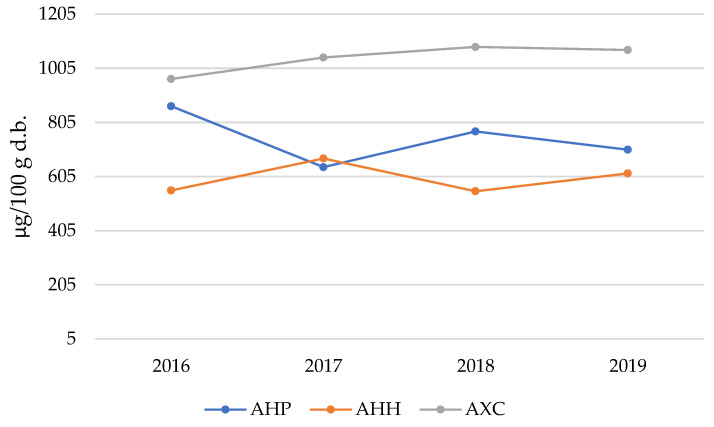
Iron (Fe) content in AHP, AHH, and AXC flower samples over the four years of study.

Among the detected metals, Fe is particularly important and ubiquitous in plant systems. It has several roles, including serving as a cofactor in enzymatic reactions, participating in electron transport chains, and facilitating metabolic processes such as photosynthesis and respiration [52,53]. Its essentiality and involvement in redox reactions make it a critical component for plant development and physiological functions. Figure 4 illustrates the trends in Fe content in *Aesculus* flowers over the four-year investigation period.

Iron occupies a transitional position between the two groups of analytes represented in Table 3, Table 4 and Table 5, with concentrations consistently below 1 mg/100 g d.b. for the two *Hippocastanaceae* cultivars (AHP and AHH) and values fluctuating near the cutoff for the AXC species.

When considering the average Fe concentration across species, the following sequence is observed:

Fe content in flowers (mg/100 g d.b.):

0.597 ± 0.044 (AHP) < 0.745 ± 0.059 (AHH) < 1.042 ± 0.056 (AXC)

The data reveal that Fe content in AXC flowers exceeds that of AHP by >75% and AHH by >40%. An intriguing comparison emerges when juxtaposing these results with the Fe content in the seeds of the same *Aesculus* cultivars [15]:

Fe content in seed flour (mg/100 g d.b.):

78.7 ± 3.2 (AHP) >> 4.41 ± 0.40 (AXC) > 1.60 ± 0.33 (AHH)

The most notable differences in Fe content between seeds and flowers can be summarized as follows:

- AHP seeds contain Fe in quantities approximately 130 times greater than the corresponding flowers, whereas the ratio is approximately 4 for AXC and only 2 for the hybrid species AHH.
- The gradient sequence for total Fe concentration is inverted between flowers and seeds. Specifically, AHP leads in seed Fe content, while AXC has the highest Fe concentration in flowers.

Another element of significant interest is Al. The distribution of the Al^3+^ cation during the observation period shows a comparable average trend for the two *Hippocastanaceae* cultivars, AHP (76.8 mg/100 g d.b.) and AHH (77.9 mg/100 g d.b.). The AXC species; however, exhibits slightly lower levels (72.7 mg/100 g d.b.), with a difference of approximately −7% compared to the *Hippocastanaceae* cultivars. From the pioneering work of Chenery [54], it is known that aluminum plays a crucial role in pigmentation processes involving floral forms enriched with anthocyanins. This is primarily due to its specific interactions with delphinidin and its glucosylated derivatives, facilitating the formation of stable complexes. Conversely, high-molecular-weight polyphenolic tannins do not appear to interfere with these interactions.

**Figure 5:**
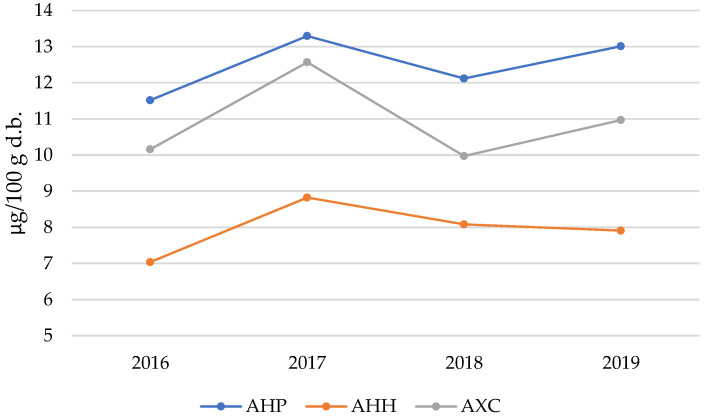
Nickel (Ni) content in AHP, AHH, and AXC flower samples over the four years of study.

Another metallic cation of particular interest is nickel (Ni^2+^). The life cycle of plant systems necessitates the involvement of at least 17 essential elements in metabolic processes, including heavy metals such as Cu, Zn, and Ni, alongside Pb and Cd [55,56]. These elements are absorbed from the physiological solutions circulating in the soil. Nickel is an essential micronutrient for plants, primarily serving as an activator of the enzyme urease. This enzyme facilitates the degradation of urea into CO_2_ and NH_3_ [57]. Unfortunately, the active site of urease, which consists of a Ni^2+^ ion, is highly susceptible to destabilization by the presence of other heavy metal cations. Such destabilization can lead to enzymatic inhibition, causing severe damage to the vegetative system. Similarly, an excess of Ni^2+^ in the soil can have deleterious effects. It tends to accumulate in the root system, impeding the absorption of other essential metals such as Fe^2+^ and Mg^2+^. For instance, the replacement of Mg^2+^ in the porphyrin center of chlorophyll by Ni^2+^ is a primary cause of reduced chlorophyll content in leaves. This phenomenon contributes to chlorosis and necrosis under stress conditions resulting from Ni overdosage and accumulation [58]. Recent studies suggest that Ni plays a crucial role in the degradation of methylglyoxal, a compound known for its potent cytotoxic properties, naturally produced during cellular metabolism. This has led researchers to hypothesize that Ni contributes significantly to the antioxidant metabolism of plants, particularly under stress or hyper-stress conditions induced by elevated Ni concentrations [59]. Figure 5 illustrates the trends in Ni content in *Aesculus* flowers over the four-year investigation period.

The trend illustrated in Figure 5 is particularly intriguing, as it reveals consistent patterns over time. The highest Ni^2+^ content is consistently observed in the AHP species, while the AHH species shows the lowest values across all years, with the AXC species positioned at intermediate levels. Perhaps the most notable observation is the nearly parallel progression of the three datasets over the time window analyzed, with a distinct peak recorded in 2017 and a subsequent decrease in 2018. These fluctuations in Ni content within the flowers of *Aesculus* are likely influenced by annual variations in weather and climatic conditions. Specifically, rainfall and the resultant changes in the acidity of the physiological solutions circulating in the soil appear to play a significant role in modulating metal concentrations.

Considering the results obtained from the analysis of seeds and flowers of *Aesculus*, it was deemed appropriate to complete the experimental framework by examining the chemical composition of the soil where the AHP, AHH, and AXC plants used in this study grow. Data collected during the investigations conducted in 2016 and 2019 are presented in Table 7. ANOVA analysis revealed no statistically significant differences among the groups for any element (*p* > 0.05 for all rows).

Some of the detected elements do not appear in Table 3, Table 4 and Table 5 because they were not detected in the floral matrices. The results for AXC obtained after several years align closely with the previous values observed for the AHP and AHH cultivars [15]. This consistency was expected, as soil sampling was conducted near the roots of the selected plants, all located on the university campus grounds. These soils, characteristic of alluvial plains, naturally contain heavy metals such as Cr and Ni, which are distributed across the area due to solid material transfer phenomena and hydro-geological processes. Similarly, the Sb content in the analyzed samples is generally consistent with the values measured by environmental monitoring laboratories and represented in the territorial maps of our region, Emilia-Romagna. Therefore, the elevated concentration of Sb in Aesculus flowers does not appear to be strictly correlated with the Sb levels measured in soil samples. Instead, it may be attributed to a metabolic process that facilitates the absorption and accumulation of this metal specifically in the floral structures of these plants.

However, an incremental increase in all measured concentrations was observed in 2019, likely attributable to anthropogenic influences, including urbanization and atmospheric deposition. While soil analysis provides essential information on the availability of elements in the rhizosphere, it is important to acknowledge that plants do not absorb metals exclusively from the soil. In addition to root uptake, elements can also be introduced into plant tissues through atmospheric deposition, foliar absorption, and surface contamination from particulate matter. These alternative pathways are particularly relevant in urban environments, where airborne pollutants originating from traffic, industrial activities, and combustion processes can settle on plant surfaces, contributing to metal accumulation. It is noteworthy that the reported concentrations of heavy metals remain well below the limits established by local, national, and European regulations for the classification of agricultural soils and public spaces such as parks and gardens, ensuring their safe use [60].

### 2.3. PCA Analysis

**Figure 6:**
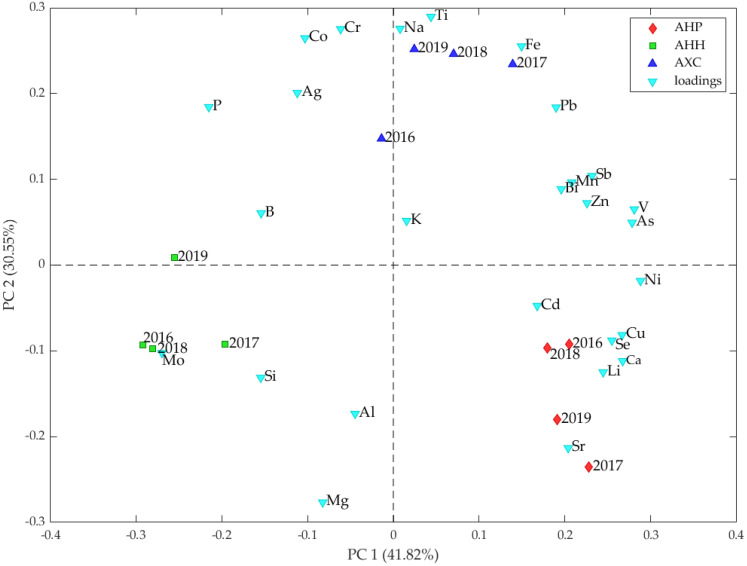
PCA Biplot.

The PCA biplot (Figure 6) illustrates the relationships among the years of sample collection, the three species (AHP, AHH, AXC), and the element concentrations in their respective flowers. The first two principal components (PC1 and PC2) explain 41.82% and 30.55% of the total variance, respectively, capturing a significant portion of the variability in the dataset.

The loading vectors represent the contributions of individual elements to the principal components. Elements such as Ti, Na, Fe, and Pb are positively correlated with both PC1 and PC2, indicating their strong influence in samples located in the top-right quadrant. These elements suggest an enrichment pattern related to AXC samples, which predominantly occupy this region of the biplot. Conversely, elements such as Mg, Al, and Si are negatively correlated with PC1 and PC2, suggesting their prominence in samples located in the bottom-left quadrant. These elements are closely associated with AHH samples. This pattern highlights consistently lower concentrations of elements associated with the top-right quadrant, reinforcing distinct compositional differences between AHH and AXC. Elements including Cu, Se, Ca, and Li cluster in the bottom-right quadrant, showing associations with AHP samples, especially in 2016 and 2018. These elements likely reflect higher concentrations in these years. Additionally, Sr appears prominently associated with the 2017 and 2019 AHP samples, suggesting temporal variations potentially linked to soil composition changes or differences in plant uptake dynamics over time. Notably, elements such as Co and P project into the upper-left quadrant, aligning more closely with specific loading patterns observed in other species. Elements such as Cd, Zn, Mn, Sb, V, Ni, and As occupy positions along the right side of the plot, indicating varying levels of correlation with PC1 and minimal association with PC2. These elements are aligned primarily with AXC samples, suggesting elevated concentrations in this species.

## 3. Materials and Methods

### 3.1. Sample Preparation and Sampling Procedures

A total of 15 trees were selected for this study, including five *Aesculus hippocastanum* L. (AHP), five hybrid *Aesculus hippocastanum* (AHH), and five *Aesculus × carnea* (AXC) specimens. These were the same trees that produced the seeds analyzed in prior studies conducted during the same period [13,15]. No chemical treatments, pesticides, or fertilizers were applied to these trees during the study period, ensuring that the elemental composition observed was not influenced by external interventions.

Between 2016 and 2019, twelve inflorescences were collected annually from each tree during the peak flowering period (late April to early May). This period coincides with the spectacular blooming of the majestic panicle inflorescences that adorn the tree-lined paths of the University Campus in Modena. To ensure the results were representative of the varied conditions across the expansive tree canopies, flowers were collected at three heights above ground (~3 m, ~4 m, and ~6 m) and along cardinal orientations (North-South and East-West).

These sampling criteria aimed to reduce variability stemming from environmental factors such as weather conditions, which could influence growth and flowering times, as well as differences in flower maturity due to varying levels of solar exposure.

At each collection, individual flowers were separated from the panicle inflorescence, with all floral components preserved except for the peduncle. The flowers were then dried, stored in sealed polyethylene (PE) bags, and kept at −18 °C until use (September 2019).

Table 8 provides detailed information on the samples collected, the analytical procedures applied, and the number of replicates performed for each sample type.

To minimize variability attributable to instrumental factors, particularly those associated with the ICP OES spectrophotometer, all experimental measurements were conducted in September 2019. This approach ensured the use of consistent reagents and operational conditions across all floral samples collected during the four-year study period (2016–2019).

Great care was taken during the preparation of the annual floral matrices to ensure that observed differences or similarities among the results reflected intrinsic sample characteristics, rather than operational inconsistencies or year-to-year variations. Conducting measurements at the end of the sampling campaign allowed for a more streamlined analysis and minimized potential errors or incidental factors that might otherwise arise over extended temporal spans.

In our previous study [15], heavy metal concentrations were determined in soil samples collected near the AHP and AHH trees. To complement these findings, composite topsoil samples (10–30 cm depth) were collected near the roots of five AXC trees concurrently with flower sampling in 2019. Soil samples were initially air-dried, powdered, and subsequently dried at 105 °C until a constant weight was achieved.

### 3.2. Proximate Analysis

The moisture content of fresh flowers was determined at each harvest year by desiccating the samples in an electric oven at 105 °C until a constant weight was achieved. The dried samples were considered as dry based (d.b.) materials for further calculations and analyses. After drying, the samples were stored in polyethylene (PE) bags at −18 °C until further use.

All experiments evaluating the content of metals and other elements were performed on materials dried and stabilized at 105 °C. Ash content was determined according to AOAC methods for similar matrices. Approximately 5 g of native flower material was incinerated and calcined in platinum crucibles at 550 °C until a constant weight was reached. The total protein content of the flower samples was determined using the Dumas combustion method, as outlined in AOAC protocols. Since plant proteins have an average nitrogen content (N %) of approximately 16%, the nitrogen content was multiplied by a factor of 6.25 (1/0.16) to calculate the total protein content.

### 3.3. Sample Preparation for Metal and Metalloid Analysis

All mineral acids and oxidants, including HNO_3_, HCl, and H_2_O_2_, were of the highest purity grade (Suprapure, Merck, Darmstadt, Germany). Plastic containers and glassware were meticulously cleaned using a 10% HNO_3_ solution, followed by thorough rinsing with deionized water (Milli-Q Plus system, Merck Millipore, Merck KGaA, Darmstadt, Germany).

For metal content determinations, approximately 1–2 g of dried flower samples were ground and digested in 4 mL of a mixture of HNO_3_ (65%) and H_2_O_2_ (30%) in a 3:2 ratio. Two replicated digestion cycles were performed for the dried flower specimens of each cultivar. Additionally, one spiked and one fortified sample were prepared to evaluate recovery factors for each genotype. The mineralized samples were diluted to a final volume of 50 mL with deionized water. The resulting solutions were clear, colorless, stable over time, and free of turbidity or precipitates, confirming the reliability of the wet digestion method.

For soil samples collected near the roots of five AXC trees, approximately 500 mg of powdered topsoil was digested with 5 mL of a 1:3 mixture of HNO_3_ (65%) and HCl (33%) using a microwave digestion system (CEM MARSX, CEM Corporation, Matthews, NC, USA). A blank and a fortified sample were included for each carousel of six Teflon-coated vessels (model XP 1500+). The microwave digestion conditions were as follows:

2 min at 250 W,
2 min at 0 W,
6 min at 250 W,
5 min at 400 W,
8 min at 550 W,
followed by an 8-min venting period.

The digested soil samples were cooled, diluted to a final volume of 50 mL, microfiltered through a 0.2 µm Teflon membrane, and directly used for ICP OES analysis.

Reference solutions for calibration were prepared using Merck ICP single standards and two multistandards containing 22 and 9 elements, respectively, at concentrations ranging from 10 to 1000 mg L⁻^1^, along with four single standards for As, B, Na, and K at 1000 mg L⁻^1^. These elements were selected as they include essential macro- and micronutrients required for plant metabolism, as well as trace elements and potential contaminants relevant to environmental and biological studies. Table S1 provides additional information on the emission wavelengths (λ), selected Limit Of Quantification (LOQ) and Linear Operative Limit (LOL), linear correlation coefficient (r), relative standard deviation (RSD) of the external calibration and recovery factor for each element determined.

### 3.4. ICP OES Determinations

A Perkin-Elmer ICP optical emission spectrometer (Optima 4200 DV, Perkin-Elmer, Waltham, MA, USA) equipped with an ultrasonic nebulizer (Cetac Technologies Inc., Omaha, NE, USA) and a CCD area detector was employed to determine the total elemental content. Selenium (Se) and mercury (Hg) were quantified using a Chemifold FIAS 400 hydride generation system (Perkin-Elmer, Waltham, MA, USA) for sample introduction into the torch.

Detailed information on the instrumental setup, assessment procedures, working methodologies, and experimental data collection has been previously reported [15].

### 3.5. Principal Component Analysis (PCA) and Data Analysis

PCA was performed using the PLS Toolbox (version 4, Eigenvector Research Inc., Wenatchee, WA, USA) running within the Matlab^®^ 2023a environment (MathWorks Inc., Natick, MA, USA). The experimental data were compared by conducting an analysis of variance (one-way ANOVA) with Tukey-Kramer honestly significant difference (HSD) post hoc testing, by using the Matlab^®^ 2023a environment. The level of significance was determined at *p* < 0.05 to see whether there were statistical differences between the mean values.

## 4. Conclusions

This study investigated the metal content in the flowers of three *Aesculus* cultivars (AHP, AHH, and AXC), which are among the most widespread ornamental trees of this family in central Europe. Metal uptake by these plants influences numerous morphological and physiological characteristics, leading to variations in both primary and secondary metabolic processes, as well as in the composition of compounds involved in these pathways.

Although the observations were limited to plants growing within the university campus of Modena, the study spans a four-year period and has yielded significant findings. The results demonstrated that while the three cultivars exhibit only slight differences in the total mineral content of their flowers, as indicated by the average ash values (AHP: 5.50%, AHH: 5.22%, AXC: 5.16%), they differ more distinctly in the elemental composition of these ashes.

Notable findings from the group of macroconstituent elements include:

- Potassium (K): The major element across all three species, showing very similar average values;
- Phosphorus (P): Occupies the second position in abundance; however, in AHP, its average content is approximately 20% lower than in AHH and AXC;
- Magnesium (Mg): AXC shows significantly lower Mg levels, approximately 4–5 times less than the two *Hyppocastanaceae* species (AHP and AHH).

One of the most intriguing findings concerns the unexpectedly high presence of antimony (Sb) in *Aesculus* flowers. Given the limited understanding of Sb’s role in floral species, these results raise important questions about its function in plant metabolism. The detected Sb concentrations suggest alternative mechanisms of metal interaction that differ from classical bioaccumulation processes. Despite extensive searches in the literature databases, no comparable data regarding the metal content in the flowers of *Aesculus* were found. However, based on the evidence collected in this study, anthropogenic and environmental contamination can reasonably be excluded as primary sources of metal absorption and bioaccumulation. Instead, the observed patterns likely reflect intrinsic characteristics and natural metabolic processes specific to these vegetative systems. These findings can have significant implications for plant physiology, environmental monitoring, and pharmacological research. They could also be relevant to studies on the nutritional value of these plant materials, as horse chestnuts are widely used as a raw material for nutritional supplements and biopharmacological applications. Future investigations should explore the functional consequences of metal accumulation and assess their potential impact on plant health, pollinator interactions, and broader ecological dynamics.

## Figures and Tables

**Table 1:** Proximate analysis results of AHP, AHH and AXC flower samples harvested in different years.

	2016	2017	2018	2019	Mean
	AHP				
Moisture %	79.1 ± 0.5 ^a^	80.1 ± 0.7 ^ab^	79.7 ± 0.6 ^ab^	80.8 ± 0.6 ^b^	79.9 ± 0.3
Proteins % (d.b.)	12.6 ± 0.7 ^a^	12.3 ± 0.8 ^a^	12.8 ± 0.5 ^a^	12.3 ± 0.7 ^a^	12.5 ± 0.3
Ashes % (d.b.)	5.67 ± 0.11 ^ac^	5.11 ± 0.12 ^b^	5.37 ± 0.09 ^ab^	5.85 ± 0.12 ^c^	5.50 ± 0.05
N % (d.b.)	2.15 ± 0.12 ^a^	2.11 ± 0.13 ^a^	2.18 ± 0.08 ^a^	2.10 ± 0.12 ^a^	2.13 ± 0.06
C % (d.b.)	45.67 ± 0.27 ^a^	45.29 ± 0.29 ^a^	45.53 ± 0.29 ^a^	45.16 ± 0.25 ^a^	45.4 ± 0.1
H % (d.b.)	5.98 ± 0.13 ^a^	5.89 ± 0.11 ^a^	5.88 ± 0.13 ^a^	5.91 ± 0.15 ^a^	5.91 ± 0.06
S % (d.b.)	0.131 ± 0.051 ^a^	0.141 ± 0.040 ^a^	0.132 ± 0.037 ^a^	0.130 ± 0.039 ^a^	0.133 ± 0.021
	AHH				
Moisture %	77.5 ± 0.7 ^a^	78.6 ± 0.9 ^a^	77.6 ± 0.6 ^a^	78.2 ± 0.9 ^a^	78.0 ± 0.4
Proteins % (d.b.)	12.8 ± 0.7 ^a^	12.6 ± 0.6 ^a^	13.2 ± 0.6 ^a^	13.2 ± 0.7 ^a^	12.9 ± 0.3
Ashes % (d.b.)	5.32 ± 0.10 ^a^	5.16 ± 0.11 ^a^	5.26 ± 0.09 ^a^	5.12 ± 0.10 ^a^	5.21 ± 0.05
N % (d.b.)	2.19 ± 0.12 ^a^	2.15 ± 0.11 ^a^	2.25 ± 0.11 ^a^	2.26 ± 0.12 ^a^	2.21 ± 0.06
C % (d.b.)	45.42 ± 0.25 ^a^	45.55 ± 0.21 ^a^	45.29 ± 0.18 ^a^	45.69 ± 0.22 ^a^	45.5 ± 0.1
H % (d.b.)	5.90 ± 0.12 ^a^	5.60 ± 0.18 ^a^	5.74 ± 0.12 ^a^	5.93 ± 0.15 ^a^	5.79 ± 0.07
S % (d.b.)	0.102 ± 0.03 ^a^	0.088 ± 0.04 ^a^	0.107 ± 0.05 ^a^	0.101 ± 0.04 ^a^	0.099 ± 0.020
	AXC				
Moisture %	77.1 ± 0.9 ^a^	77.6 ± 1.0 ^a^	78.2 ± 0.7 ^a^	77.9 ± 0.9 ^a^	77.7 ± 0.4
Proteins % (d.b.)	12.5 ± 0.7 ^a^	12.2 ± 0.8 ^a^	12.9 ± 0.5 ^a^	12.8 ± 0.7 ^a^	12.6 ± 0.3
Ashes % (d.b.)	5.01 ± 0.11 ^a^	5.14 ± 0.09 ^ab^	5.12 ± 0.11 ^a^	5.37 ± 0.10 ^b^	5.16 ± 0.05
N % (d.b.)	2.13 ± 0.12 ^a^	2.08 ± 0.13 ^a^	2.21 ± 0.09 ^a^	2.18 ± 0.12 ^a^	2.15 ± 0.06
C % (d.b.)	44.97 ± 0.37 ^a^	44.51 ± 0.39 ^a^	44.78 ± 0.26 ^a^	44.39 ± 0.27 ^a^	44.7 ± 0.2
H % (d.b.)	5.68 ± 0.11 ^a^	5.79 ± 0.10 ^a^	5.72 ± 0.09 ^a^	5.81 ± 0.11 ^a^	5.75 ± 0.05
S % (d.b.)	0.100 ± 0.04 ^a^	0.089 ± 0.03 ^a^	0.091 ± 0.04 ^a^	0.100 ± 0.02 ^a^	0.095 ± 0.017

Data are expressed as mean ± standard deviation of three replicates. Difference between means indicated by the same letters are not statistically significant (*p* < 0.05).

**Table 2:** Comparison of the mineral fraction content between some *Aesculus* seed flours [15] and flowers samples of different botanical origins (AHP, AHH, AXC) harvested in different years.

	2016	2017	2018	2019
	AHP			
Ashes % (d.b.) of seeds	2.48 ± 0.14 ^a^	2.44 ± 0.16 ^a^	2.50 ± 0.16 ^a^	2.46 ± 0.15 ^a^
Ashes % (d.b.) of flowers	5.67 ± 0.11 ^ac^	5.11 ± 0.12 ^b^	5.37 ± 0.09 ^ab^	5.85 ± 0.12 ^c^
	AHH			
Ashes % (d.b.) of seeds	2.15 ± 0.11 ^a^	2.11 ± 0.12 ^a^	2.13 ± 0.11 ^a^	2.21 ± 0.10 ^a^
Ashes % (d.b.) of flowers	5.32 ± 0.10 ^a^	5.16 ± 0.11 ^a^	5.26 ± 0.09 ^a^	5.12 ± 0.10 ^a^
	AXC			
Ashes % (d.b.) of seeds	3.05 ± 0.09 ^a^	3.14 ± 0.10 ^a^	3.09 ± 0.08 ^a^	3.12 ± 0.08 ^a^
Ashes % (d.b.) of flowers	5.01 ± 0.11 ^a^	5.14 ± 0.09 ^ab^	5.12 ± 0.11 ^a^	5.37 ± 0.10 ^b^

Data are expressed as mean ± standard deviation of three replicates. Difference between means indicated by the same letters are not statistically significant (*p* < 0.05).

**Table 3:** Element concentrations in flours from AHP, collected in different years.

	2016	2017	2018	2019
	mg/100 g d.b.			
Ca	49.9 ± 2.1	48.8 ± 1.3	47.8 ± 3.1	48.9 ± 3.4
Fe	0.865 ± 0.063 ^a^	0.640 ± 0.061 ^bc^	0.771 ± 0.040 ^ac^	0.704 ± 0.068 ^bc^
K	1388 ± 73	1235 ± 97	1264 ± 59	1378 ± 105
Mg	71.4 ± 3.9	74.3 ± 3.4	70.3 ± 4.0	76.5 ± 5.6
Na	10.8 ± 1.9	11.6 ± 2.2	12.0 ± 1.0	11.1 ± 1.4
P	708 ± 87	693 ± 55	679 ± 61	713 ± 76
Sb	2.66 ± 0.21 ^a^	6.46 ± 0.42 ^b^	3.86 ± 0.32 ^c^	4.79 ± 0.35 ^d^
Si	18.4 ± 2.7	21.7 ± 2.1	19.3 ± 1.8	22.8 ± 2.0
	μg/100 g d.b.			
Ag	1.04 ± 0.17	0.86 ± 0.28	1.05 ± 0.11	0.99 ± 0.12
Al	72.3 ± 8.5	81.4 ± 4.4	68.5 ± 11.1	85.0 ± 2.7
As	46.2 ± 4.5	39.9 ± 4.0	42.6 ± 4.8	48.4 ± 4.0
B	159 ± 23 ^a^	147 ± 31 ^a^	197 ± 19 ^b^	142 ± 14 ^a^
Bi	12.1 ± 1.0 ^a^	9.28 ± 0.88 ^b^	9.79 ± 0.89 ^b^	11.2 ± 1.7 ^a^
Cd	38.6 ± 5.8	38.8 ± 3.5	40.1 ± 2.5	35.1 ± 2.1
Co	0.87 ± 0.07 ^a^	0.32 ± 0.04 ^b^	0.69 ± 0.03 ^c^	0.59 ± 0.07 ^c^
Cr	113 ± 9 ^a^	78.3 ± 7.1 ^b^	133 ± 11 ^c^	128 ± 9 ^ac^
Cu	159 ± 12 ^a^	232 ± 24 ^b^	185 ± 23 ^ab^	206 ± 18 ^ab^
Li	24.3 ± 3.5	20.4 ± 2.0	21.5 ± 2.4	23.6 ± 2.9
Mn	248 ± 14	217 ± 16	252 ± 25	234 ± 20
Mo	4.35 ± 0.41 ^a^	2.69 ± 0.23 ^b^	4.17 ± 0.19 ^a^	2.89 ± 0.23 ^b^
Ni	11.5 ± 2.6	13.3 ± 3.2	12.1 ± 1.1	13.0 ± 0.7
Pb	11.8 ± 2.9 ^a^	20.8 ± 2.4 ^bc^	17.6 ± 0.9 ^ac^	23.4 ± 2.3 ^bc^
Se	33.7 ± 5.5	42.7 ± 3.9	38.2 ± 4.7	36.2 ± 2.9
Sr	169 ± 33	184 ± 34	167 ± 12	173 ± 13
Ti	17.2 ± 4.8 ^a^	1.69 ± 0.28 ^b^	10.6 ± 1.9 ^ac^	5.06 ± 0.75 ^bc^
V	158 ± 22	161 ± 20	149 ± 15	142 ± 19
Zn	373 ± 27 ^a^	295 ± 27 ^bc^	341 ± 19 ^ac^	287 ± 22 ^bc^

Data are expressed as mean ± standard deviation of three replicates. Different between means indicated by the same letters are not statistically significant (*p* < 0.05). If no letters are present, the differences between means are not significant.

**Table 4:** Element concentrations in flours from AHH, collected in different years.

	2016	2017	2018	2019
	mg/100 g d.b.			
Ca	34.2 ± 1.8	37.9 ± 3.4	39.9 ± 3.5	33.9 ± 2.5
Fe	0.553 ± 0.045 ^a^	0.671 ± 0.053 ^b^	0.550 ± 0.035 ^a^	0.616 ± 0.043 ^ab^
K	1314 ± 67	1386 ± 119	1283 ± 93	1338 ± 147
Mg	79.3 ± 3.1	78.5 ± 5.3	79.9 ± 3.8	78.1 ± 4.9
Na	11.5 ± 0.8 ^a^	10.1 ± 1.2 ^a^	12.1 ± 2.2 ^a^	17.2 ± 2.2 ^b^
P	842 ± 49	824 ± 54	879 ± 97	867 ± 44
Sb	1.72 ± 0.46 ^a^	1.38 ± 0.14 ^bc^	1.57 ± 0.10 ^ac^	1.29 ± 0.11 ^bc^
Si	24.0 ± 1.7	20.5 ± 2.2	23.8 ± 2.1	21.2 ± 2.3
	μg/100 g d.b.			
Ag	1.12 ± 0.14 ^ab^	0.89 ± 0.14 ^a^	1.14 ± 0.27 ^ab^	1.29 ± 0.12 ^b^
Al	81.4 ± 5.7	76.5 ± 7.9	79.4 ± 8.8	74.2 ± 11.1
As	24.1 ± 2.0 ^a^	18.0 ± 1.2 ^bc^	22.1 ± 1.9 ^ac^	19.5 ± 2.7 ^ac^
B	197 ± 22 ^a^	145 ± 29 ^b^	199 ± 23 ^a^	204 ± 17 ^a^
Bi	8.02 ± 0.85 ^a^	9.02 ± 1.45 ^a^	7.24 ± 0.87 ^b^	8.29 ± 1.63 ^ab^
Cd	30.9 ± 2.8 ^a^	33.2 ± 2.8 ^ab^	31.3 ± 3.0 ^a^	39.2 ± 3.2 ^b^
Co	1.48 ± 0.11	1.62 ± 0.25	1.54 ± 0.16	1.79 ± 0.20
Cr	179 ± 12 ^a^	151 ± 13 ^b^	144 ± 13 ^b^	159 ± 12 ^ab^
Cu	105 ± 11	96.8 ± 6.2	91.6 ± 6.5	102 ± 10
Li	14.5 ± 1.7 ^a^	10.8 ± 1.4 ^b^	12.4 ± 1.2 ^ab^	11.1 ± 1.0 ^ab^
Mn	155 ± 14 ^a^	180 ± 13 ^ab^	175 ± 12 ^ab^	193 ± 16 ^b^
Mo	14.2 ± 1.5	14.7 ± 1.7	12.7 ± 1.6	12.6 ± 1.3
Ni	7.04 ± 1.25	8.82 ± 1.23	8.08 ± 0.87	7.91 ± 0.95
Pb	7.90 ± 1.49	10.7 ± 1.0	7.84 ± 1.4	9.23 ± 0.56
Se	18.5 ± 1.8 ^a^	24.4 ± 1.7 ^b^	22.5 ± 2.1 ^ab^	20.0 ± 2.4 ^ab^
Sr	109 ± 10	101 ± 4	106 ± 8	101 ± 7
Ti	11.1 ± 1.3	12.3 ± 2.0	10.2 ± 1.7	11.3 ± 1.3
V	102 ± 15	96.4 ± 10.7	98.0 ± 5.1	104 ± 8
Zn	235 ± 14	266 ± 19	219 ± 14	253 ± 15

Data are expressed as mean ± standard deviation of three replicates. Different between means indicated by the same letters are not statistically significant (*p* < 0.05). If no letters are present, the differences between means are not significant.

**Table 5:** Element concentrations in flours from AXC, collected in different years.

	2016	2017	2018	2019
	mg/100 g d.b.			
Ca	39.1 ± 1.9	43.6 ± 2.2	40.9 ± 3.6	38.7 ± 2.9
Fe	0.965 ± 0.023	1.045 ± 0.057	1.084 ± 0.052	1.073 ± 0.079
K	1271 ± 69 ^a^	1385 ± 82 ^ab^	1509 ± 77 ^b^	1190 ± 111 ^a^
Mg	14.8 ± 2.3	19.9 ± 2.5	16.8 ± 1.9	15.4 ± 1.6
Na	18.5 ± 1.4	19.5 ± 1.3	18.8 ± 1.8	18.0 ± 1.9
P	826 ± 55	854 ± 68	810 ± 86	905 ± 80
Sb	5.26 ± 0.36	5.66 ± 0.27	5.35 ± 0.43	5.09 ± 0.31
Si	19.9 ± 1.6	20.5 ± 1.9	18.1 ± 1.6	20.9 ± 1.8
	μg/100 g d.b.			
Ag	1.14 ± 0.18	1.13 ± 0.12	1.13 ± 0.19	1.29 ± 0.15
Al	71.6 ± 6.3	75.7 ± 6.7	72.4 ± 5.1	71.2 ± 6.3
As	40.7 ± 2.6	42.2 ± 3.1	39.4 ± 3.8	40.9 ± 3.8
B	153 ± 14 ^a^	158 ± 18 ^a^	186 ± 27 ^b^	188 ± 19 ^b^
Bi	9.20 ± 1.06 ^a^	9.42 ± 1.00 ^a^	10.5 ± 1.0 ^a^	13.1 ± 1.4 ^b^
Cd	31.0 ± 1.4 ^a^	40.2 ± 3.4 ^b^	34.4 ± 3.2 ^ab^	31.6 ± 2.8 ^a^
Co	1.96 ± 0.15 ^a^	2.59 ± 0.37 ^bc^	2.70 ± 0.23 ^b^	1.85 ± 0.26 ^a^
Cr	214 ± 16 ^a^	244 ± 18 ^b^	198 ± 20 ^a^	232 ± 21 ^b^
Cu	107 ± 12 ^a^	178 ± 9 ^b^	153 ± 15 ^bc^	126 ± 11 ^ac^
Li	16.5 ± 2.3	17.1 ± 3.5	13.9 ± 2.1	12.5 ± 1.3
Mn	177 ± 6 ^a^	274 ± 11 ^b^	315 ± 19 ^c^	193 ± 15 ^a^
Mo	2.69 ± 0.42	2.58 ± 0.32	2.89 ± 0.28	2.68 ± 0.22
Ni	10.2 ± 2.5	12.6 ± 2.7	9.97 ± 0.76	11.0 ± 1.0
Pb	20.6 ± 1.7 ^a^	35.3 ± 3.4 ^b^	23.9 ± 2.5 ^ac^	31.0 ± 3.0 ^bc^
Se	33.8 ± 2.2 ^a^	24.6 ± 2.2 ^b^	27.9 ± 2.2 ^ac^	29.8 ± 3.0 ^ac^
Sr	95.1 ± 11.0	98.1 ± 9.9	101 ± 10	100 ± 12
Ti	24.6 ± 2.1 ^a^	40.6 ± 3.0 ^b^	43.7 ± 2.9 ^b^	38.3 ± 3.4 ^b^
V	130 ± 10	174 ± 32	151 ± 11	135 ± 11
Zn	252 ± 11 ^a^	334 ± 22 ^bc^	287 ± 30 ^ac^	363 ± 26 ^b^

Data are expressed as mean ± standard deviation of three replicates. Different between means indicated by the same letters are not statistically significant (*p* < 0.05). If no letters are present, the differences between means are not significant.

**Table 6:** Mean values of the content of N, P, and K in the flowers of *Aesculus* trees for four years (2016–2019) of observations.

	AHP	AHH	AXC
N %	2.13 ± 0.06 ^a^	2.21 ± 0.06 ^a^	2.15 ± 0.06 ^a^
P %	0.698 ± 0.03 ^a^	0.853 ± 0.03 ^b^	0.849 ± 0.04 ^b^
K %	1.316 ± 0.04 ^a^	1.330 ± 0.05 ^b^	1.339 ± 0.04 ^b^
N %/P %	3.066 ± 0.178 ^a^	2.591 ± 0.121 ^b^	2.535 ± 0.130 ^b^
N %/K %	1.626 ± 0.070 ^a^	1.662 ± 0.082 ^a^	1.606 ± 0.068 ^a^
P %/K %	0.5304 ± 0.0319 ^a^	0.6414 ± 0.0360 ^b^	0.6341 ± 0.0341 ^b^

Data are expressed as mean ± standard deviation of three replicates. Different between means indicated by the same letters are not statistically significant (*p* < 0.05).

**Table 7:** Concentrations of some elements (mg/100 g d.b.) in soil samples.

	AHP *	AHH *	AXC ^#^
Ag	0.0002 ± 0.0002	0.0004 ± 0.0004	0.0004 ± 0.0004
Al	5812 ± 93	5855 ± 124	5827 ± 106
As	0.75 ± 0.07	0.81 ± 0.10	0.84 ± 0.13
B	0.049 ± 0.006	0.047 ± 0.007	0.051 ± 0.009
Bi	1.83 ± 0.10	1.84 ± 0.07	1.95 ± 0.12
Ca	3840 ± 387	4009 ± 236	3913 ± 325
Cd	0.20 ± 0.02	0.19 ± 0.02	0.23 ± 0.03
Co	2.37 ± 0.14	2.39 ± 0.18	2.65 ± 0.15
Cr	9.30 ± 0.16	9.34 ± 0.16	9.67 ± 0.19
Cu	10.70 ± 0.49	10.42 ± 0.71	10.8 ± 0.7
Fe	3165 ± 174	3195 ± 132	3078 ± 165
Ga	2.89 ± 0.15	2.89 ± 0.14	2.82 ± 0.12
In	1.38 ± 0.26	1.28 ± 0.12	1.31 ± 0.17
K	2918 ± 148	2937 ± 155	2996 ± 174
Li	1.83 ± 0.12	1.82 ± 0.11	1.95 ± 0.12
Mg	430.3 ± 32.0	419.9 ± 49.6	452.5 ± 54.3
Mn	122.9 ± 6.1	123.5 ± 7.8	121.1 ± 5.9
Mo	1.05 ± 0.15	1.16 ± 0.11	1.08 ± 0.09
Na	2947 ± 144	2986 ± 109	2866 ± 121
Ni	5.90 ± 0.22	5.82 ± 0.27	5.71 ± 0.30
P	55.8 ± 2.9	56.1 ± 2.3	55.2 ± 2.7
Pb	10.59 ± 0.63	10.32 ± 0.69	10.7 ± 0.7
Sb	0.113 ± 0.012	0.116 ± 0.011	0.103 ± 0.009
Se	0.042 ± 0.004	0.044 ± 0.004	0.041 ± 0.005
Si (%)	20.7 ± 0.2	20.8 ± 0.2	20.7 ± 0.2
Sr	31.4 ± 2.4	32.2 ± 2.6	31.0 ± 2.8
Ti	1.62 ± 0.14	1.69 ± 0.13	1.64 ± 0.09
Tl	0.033 ± 0.009	0.038 ± 0.008	0.035 ± 0.014
V	3.47 ± 0.23	3.36 ± 0.13	3.37 ± 0.21
Zn	14.8 ± 1.2	14.9 ± 0.8	14.3 ± 1.0

Data are expressed as mean ± standard deviation of three replicates and are representative of the growing soil trees for the three varieties of *A. hippocastanum* (AHP, AHH, and AXC); * Data collected in the year 2016; ^#^ Data collected in the year 2019.

**Table 8:** Work plant for independent flower samples and sampling procedures for ICP OES determination, applied to different horse-chestnut specimens (AHP, AHH, and AXC) from 2016 to 2019.

	Year 2016	Year 2017	Year 2018	Year 2019
N° of trees—AHP	5	5	5	5
N° of trees—AHH	5	5	5	5
N° of trees—AXC	5	5	5	5
N° of independent replicates for each tree	2 +1 spiked +1 fortified	2 +1 spiked +1 fortified	2 +1 spiked +1 fortified	2 +1 spiked +1 fortified
Total n° of replicates for each variety (AHP, AHH and AXC)	10 +5 spiked +5 fortified	10 +5 spiked +5 fortified	10 +5 spiked +5 fortified	10 +5 spiked +5 fortified

## Data Availability

Data are contained within the article.

## Supplementary Materials

The following supporting information can be downloaded at: https://www.mdpi.com/article/10.3390/molecules30040908/s1, Table S1: Emission wavelengths (λ), selected Limit Of Quantification (LOQ) and Linear Operative Limit (LOL), linear correlation coefficient (r), relative standard deviation (RSD) of the external calibration and recovery factor for each element determined.

## References

[1] Pereira A.G., Cassani L., Liu C., Li N., Chamorro F., Barreira J.C.M., Simal-Gandara J., Prieto M.A. (2023). Camellia Japonica Flowers as a Source of Nutritional and Bioactive Compounds. Foods.

[2] Lancellotti L., Sighinolfi S., Ulrici A., Maletti L., Durante C., Marchetti A., Tassi L. (2021). Tracing Geographical Origin of Lambrusco PDO Wines Using Isotope Ratios of Oxygen, Boron, Strontium, Lead and Their Elemental Concentration. Curr. Res. Food Sci..

[3] D’Eusanio V., Genua F., Marchetti A., Morelli L., Tassi L. (2023). Exploring the Mineral Composition of Grapevine Canes for Wood Chip Applications in Alcoholic Beverage Production to Enhance Viticulture Sustainability. Beverages.

[4] Uchimiya M., Bannon D., Nakanishi H., McBride M.B., Williams M.A., Yoshihara T. (2020). Chemical Speciation, Plant Uptake, and Toxicity of Heavy Metals in Agricultural Soils. J. Agric. Food Chem..

[5] Lancellotti L., D’Eusanio V., Morelli L., Truzzi E., Marchetti A., Tassi L. (2025). Use of Compound Specific Isotope Analysis Approach to Monitor the Aging Process of Italian Balsamic Vinegars. Curr. Res. Food Sci..

[6] Čukanović J., Tešević V., Jadranin M., Ljubojević M., Mladenović E., Kostić S. (2020). Horse Chestnut (*Aesculus hippocastanum* L.) Seed Fatty Acids, Flavonoids and Heavy Metals Plasticity to Different Urban Environments. Biochem. Syst. Ecol..

[7] Gagic T., Knez Z., Skerget M. (2021). Subcritical Water Extraction of Horse Chestnut (*Aesculus hippocastanum*) Tree Parts. J. Serbian Chem. Soc..

[8] Weryszko-Chmielewska E., Tietze M., Michonska M. (2012). Ecological Features of the Flowers of *Aesculus hippocastanum* L. and Characteristics of Aesculus L. Pollen Seasons under the Conditions of Central-Eastern Poland. Acta Agrobot..

[9] Vasilevskaya N. (2022). Pollution of the Environment and Pollen: A Review. Stresses.

[10] Thomas P.A., Alhamd O., Iszkuło G., Dering M., Mukassabi T.A. (2019). Biological Flora of the British Isles: Aesculus Hippocastanum. J. Ecol..

[11] Patel K.S., Sharma R., Dahariya N.S., Yadav A., Blazhev B., Matini L., Hoinkis J. (2015). Heavy Metal Contamination of Tree Leaves. Am. J. Anal. Chem..

[12] Baraldi C., Bodecchi L.M., Cocchi M., Durante C., Ferrari G., Foca G., Grandi M., Marchetti A., Tassi L., Ulrici A. (2007). Chemical Composition and Characterisation of Seeds from Two Varieties (Pure and Hybrid) of Aesculus Hippocastanum. Food Chem..

[13] Baraldi C., Foca G., Maletti L., Marchetti A., Roncaglia F., Sighinolfi S., Tassi L. (2020). Red Horse-Chestnut Seeds of Aesculus × Carnea. Nuts and Seeds in Health and Disease Prevention.

[14] Foca G., Ulrici A., Cocchi M., Durante C., Vigni M.L., Marchetti A., Sighinolfi S., Tassi L. (2011). Seeds of Horse Chestnut (*Aesculus hippocastanum* L.) and Their Possible Utilization for Human Consumption. Nuts and Seeds in Health and Disease Prevention.

[15] Durante C., Cocchi M., Lancellotti L., Maletti L., Marchetti A., Roncaglia F., Sighinolfi S., Tassi L. (2021). Analytical Concentrations of Some Elements in Seeds and Crude Extracts from Aesculus Hippocastanum, by ICP-OES Technique. Agronomy.

[16] Senol Deniz F.S., Orhan I.E., Duman H. (2021). Profiling Cosmeceutical Effects of Various Herbal Extracts through Elastase, Collagenase, Tyrosinase Inhibitory and Antioxidant Assays. Phytochem. Lett..

[17] Sirtori C.R. (2001). Aescin: Pharmacology, Pharmacokinetics and Therapeutic Profile. Pharmacol. Res..

[18] Dudek-Makuch M., Studzińska-Sroka E. (2015). Horse Chestnut–Efficacy and Safety in Chronic Venous Insufficiency: An Overview. Rev. Bras. Farmacogn..

[19] Dudek-Makuch M., Matławska I. (2011). Flavonoids from the Flowers of Aesculus Hippocastanum. Acta Pol. Pharm..

[20] Dudek-Makuch M., Matławska I. (2013). Coumarins in Horse Chestnut Flowers: Isolation and Quantification by UPLC Method. Acta Pol. Pharm..

[21] Dudek-Makuch M., Studzińska-Sroka E., Korybalska K., Czepulis N., Łuczak J., Rutkowski R., Marczak Ł., Długaszewska J., Grabowska K., Stobiecki M. (2019). Biological Activity of *Aesculus hippocastanum* Flower Extracts on Vascular Endothelial Cells Cultured in Vitro. Phytochem. Lett..

[22] Deli J., Matus Z., Tóth G. (2000). Comparative Study on the Carotenoid Composition in the Buds and Flowers of differentAesculus Species. Chromatographia.

[23] Owczarek A., Kołodziejczyk-Czepas J., Marczuk P., Siwek J., Wąsowicz K., Olszewska M. (2021). Bioactivity Potential of *Aesculus hippocastanum* L. Flower: Phytochemical Profile, Antiradical Capacity and Protective Effects on Human Plasma Components under Oxidative/Nitrative Stress In Vitro. Pharmaceuticals.

[24] Bobaev I.D., Kh I.S., Normatov A.M., Khujamshukurov N.A. (2021). Component Composition of Essential Oil of Common Horse Chestnut Flowers (Hippocastanum) Growing in Tashkent Region. Talim Va Rivojlanish Tahlili Onlayn Ilmiy Jurnali.

[25] Idris S., Mishra A., Khushtar M. (2020). Phytochemical, Ethanomedicinal and Pharmacological Applications of Escin from *Aesculus hippocastanum* L. towards Future Medicine. J. Basic Clin. Physiol. Pharmacol..

[26] D’Eusanio V., Malferrari D., Marchetti A., Roncaglia F., Tassi L. (2023). Waste By-Product of Grape Seed Oil Production: Chemical Characterization for Use as a Food and Feed Supplement. Life.

[27] D’Eusanio V., Genua F., Marchetti A., Morelli L., Tassi L. (2023). Characterization of Some Stilbenoids Extracted from Two Cultivars of Lambrusco—Vitis Vinifera Species: An Opportunity to Valorize Pruning Canes for a More Sustainable Viticulture. Molecules.

[28] D’Eusanio V., Marchetti A., Rivi M., Morelli L., Scarponi P., Forti L., Tassi L. (2025). Mineral Composition Analysis of Red Horse-Chestnut (Aesculus × Carnea) Seeds and Hydroalcoholic Crude Extract Using ICP OES. Molecules.

[29] Boboev I., Iskhakova S., Normatov A., Khujamshukurov N., Otajonov A. (2024). Chemical Composition and Antimicrobial Activity of Essential Oils from Horse Chestnut Flowers. E3S Web Conf..

[30] Templeton D.W., Laurens L.M.L. (2015). Nitrogen-to-Protein Conversion Factors Revisited for Applications of Microalgal Biomass Conversion to Food, Feed and Fuel. Algal Res..

[31] Güsewell S. (2004). N: P Ratios in Terrestrial Plants: Variation and Functional Significance. New Phytol..

[32] Güsewell S., Koerselman W., Verhoeven J.T.A. (2003). Biomass N:P Ratios as Indicators of Nutrient Limitation for Plant Populations in Wetlands. Ecol. Appl..

[33] Sardans J., Rivas-Ubach A., Peñuelas J. (2012). The C:N:P Stoichiometry of Organisms and Ecosystems in a Changing World: A Review and Perspectives. Perspect. Plant Ecol. Evol. Syst..

[34] Wang M., Moore T.R. (2014). Carbon, Nitrogen, Phosphorus, and Potassium Stoichiometry in an Ombrotrophic Peatland Reflects Plant Functional Type. Ecosystems.

[35] Raghothama K.G. (2005). Phosphorus and Plant Nutrition: An Overview. Phosphorus: Agriculture and the Environment.

[36] Hasanuzzaman M., Bhuyan M.H.M.B., Nahar K., Hossain M.S., Mahmud J.A., Hossen M.S., Masud A.A.C., Moumita, Fujita M. (2018). Potassium: A Vital Regulator of Plant Responses and Tolerance to Abiotic Stresses. Agronomy.

[37] Epstein E. (2009). Silicon: Its Manifold Roles in Plants. Ann. Appl. Biol..

[38] Strout G., Russell S.D., Pulsifer D.P., Erten S., Lakhtakia A., Lee D.W. (2013). Silica Nanoparticles Aid in Structural Leaf Coloration in the Malaysian Tropical Rainforest Understorey Herb Mapania Caudata. Ann. Bot..

[39] Cooke J., Leishman M.R. (2016). Consistent Alleviation of Abiotic Stress with Silicon Addition: A Meta-Analysis. Funct. Ecol..

[40] Whalen N.S., Hunt T.C., Erickson G.M. (2023). Evapotranspiration-Linked Silica Deposition in a Basal Tracheophyte Plant (Lycopodiaceae: Lycopodiella Alopecuroides): Implications for the Evolutionary Origins of Phytoliths. New Phytol..

[41] Saerens A., Ghosh M., Verdonck J., Godderis L. (2019). Risk of Cancer for Workers Exposed to Antimony Compounds: A Systematic Review. Int. J. Environ. Res. Public. Health.

[42] Kowalczyk E., Givelet L., Amlund H., Sloth J.J., Hansen M. (2022). Risk Assessment of Rare Earth Elements, Antimony, Barium, Boron, Lithium, Tellurium, Thallium and Vanadium in Teas. EFSA J..

[43] Stroud R.M., Nollert P., Miercke L. (2003). The Glycerol Facilitator GlpF Its Aquaporin Family of Channels, and Their Selectivity. Adv. Protein Chem..

[44] Agre P., King L.S., Yasui M., Guggino W.B., Ottersen O.P., Fujiyoshi Y., Engel A., Nielsen S. (2002). Aquaporin Water Channels--from Atomic Structure to Clinical Medicine. J. Physiol..

[45] Preston G.M., Agre P. (1991). Isolation of the cDNA for Erythrocyte Integral Membrane Protein of 28 Kilodaltons: Member of an Ancient Channel Family. Proc. Natl. Acad. Sci. USA.

[46] Bhattacharjee H., Mukhopadhyay R., Thiyagarajan S., Rosen B.P. (2008). Aquaglyceroporins: Ancient Channels for Metalloids. J. Biol..

[47] Tisarum R., Lessl J.T., Dong X., de Oliveira L.M., Rathinasabapathi B., Ma L.Q. (2014). Antimony Uptake, Efflux and Speciation in Arsenic Hyperaccumulator Pteris Vittata. Environ. Pollut. Barking Essex 1987.

[48] Feng R., Wei C., Tu S., Tang S., Wu F. (2011). Simultaneous Hyperaccumulation of Arsenic and Antimony in Cretan Brake Fern: Evidence of Plant Uptake and Subcellular Distributions. Microchem. J..

[49] Feng R., Wei C., Tu S., Ding Y., Wang R., Guo J. (2013). The Uptake and Detoxification of Antimony by Plants: A Review. Environ. Exp. Bot..

[50] Zhuang W., Lai X., Wang Q., Liu Y., Chen Q., Liu C. (2018). Distribution Characteristics, Sources and Ecological Risk of Antimony in the Surface Sediments of Changjiang Estuary and the Adjacent Sea, East China. Mar. Pollut. Bull..

[51] Sharma A., Ramakrishnan M., Khanna K., Landi M., Prasad R., Bhardwaj R., Zheng B. (2022). Brassinosteroids and Metalloids: Regulation of Plant Biology. J. Hazard. Mater..

[52] Briat J.-F., Curie C., Gaymard F. (2007). Iron Utilization and Metabolism in Plants. Curr. Opin. Plant Biol..

[53] Rout G.R., Sahoo S. (2015). Role of Iron in Plant Growth and Metabolism. Rev. Agric. Sci..

[54] Chenery E.M. (1948). Aluminium in Plants and Its Relation to Plant Pigments. Ann. Bot..

[55] Broadley M., Brown P., Cakmak I., Ma J.F., Rengel Z., Zhao F., Marschner P. (2012). Chapter 8—Beneficial Elements. Marschner’s Mineral Nutrition of Higher Plants (Third Edition).

[56] Reeves R., Baker A. (2000). Metal-Accumulating Plants. Phytoremediation of Toxic Metals: Using Plants to Clean Up the Environment.

[57] Fabiano C.C., Tezotto T., Favarin J.L., Polacco J.C., Mazzafera P. (2015). Essentiality of Nickel in Plants: A Role in Plant Stresses. Front. Plant Sci..

[58] Helaoui S., Mkhinini M., Boughattas I., Alphonse V., Giusti-Miller S., Livet A., Banni M., Bousserrhine N. (2020). Assessment of Changes on Rhizospheric Soil Microbial Biomass, Enzymes Activities and Bacterial Functional Diversity under Nickel Stress in Presence of Alfafa Plants. Soil Sediment Contam. Int. J..

[59] Bhardwaj I., Garg N. (2023). Phytohormones and Arbuscular Mycorrhizal *Rhizoglomus Intraradices* Together Modulate Defense Mechanisms in Mungbean to Reduce Ni Toxicity. Rhizosphere.

[60] ARPAE-Agenzia Regionale per La Prevenzione, L’ambiente e L’energia Dell’Emilia-Romagna. Metal Content in Soil. https://webbook.arpae.it/indicatore/Contenuto-di-metalli-nel-suolo-00001/?espandi=Scopo.

