# Supplementary material for: Long-Term Variability in the Content of Some Metals and Metalloids in Aesculus Flowers: A Four-Year Study Using ICP OES and PCA Analysis
Source: Molecules. 2025 Feb 15;30(4):908. doi: 10.3390/molecules30040908 (PMC11858342; doi:10.3390/molecules30040908)
Supplement: Supplementary file 1 [file molecules-30-00908-s001.zip › molecules-3431600-supplementary.pdf]

Table S1. Emission wavelengths (λ), selected Limit Of Quantification (LOQ) and Linear Operative Limit (LOL), linear correlation coefficient (r), relative standard deviation (RSD) of the external calibration and recovery factor for each element determined.

Element	λ (nm)	LOQ ÷ LOL (mg L^{-1})	r	RSD%	Recovery%
Ag	328.068	0.005 ÷ 50	0.9995	4.9	96
Al	308.215<br>394.401	0.005 ÷ 20	0.9997	5.6	105
As	188.980<br>193.696	0.01 ÷ 50	0.9982	6.3	106
B	249.678<br>249.772	0.015 ÷ 50	0.9975	6.2	97
Bi	223.061<br>306.766	0.01 ÷ 10	0.9968	8.1	96
Ca	317.933<br>396.847	0.01 ÷ 100	0.9998	5.4	100
Cd	214.440<br>226.502	0.005 ÷ 10	0.9990	4.9	93
Co	228.616	0.005 ÷ 10	0.9979	3.5	94
Cr	267.716<br>283.563	0.005 ÷ 20	0.9978	11.3	98
Cu	324.752<br>327.393	0.005 ÷ 30	0.9999	5.0	99
Fe	238.204<br>259.939	0.005 ÷ 100	0.9997	4.5	97
Ga	294.364	0.03 ÷ 10	0.9983	3.7	98
In	325.609	0.03 ÷ 10	0.9990	2.8	99
K	766.490	0.01 ÷ 200	0.9995	7.2	94
Li	610.365<br>670.783	0.001 ÷ 50	0.9996	3.1	103
Mg	280.271<br>285.213	0.01 ÷ 200	0.9997	6.2	95
Mn	257.610<br>259.372	0.005 ÷ 40	0.9999	5.4	100

Mo	202.031<br>203.845	0.005 ÷ 30	0.9987	1.8	106
Na	589.592<br>330.237	0.01 ÷ 100	0.9998	4.6	95
Ni	231.604<br>341.476	0.005 ÷ 50	0.9999	6.7	102
P	213.617<br>214.914	10 ÷ 2000	0.9989	9.2	95
Pb	220.353<br>261.458	0.03 ÷ 40	0.9986	8.2	94
Sb	206.838<br>217.582	0.005 ÷ 30	0.9982	3.5	98
Se	196.026<br>203.985	0.01 ÷ 50	0.9998	5.1	96
Si	251.612<br>288.158	0.1 ÷ 10	0.9992	8.3	94
Sr	407.771<br>421.552	0.01 ÷ 50	0.9996	7.0	95
Ti	334.940<br>336.121	0.005 ÷ 100	0.9979	4.2	94
Tl	351.923	0.05 ÷ 50	0.9983	3.9	94
V	309.310	0.005 ÷ 40	0.9988	5.0	98
Zn	202.548<br>213.857	0.05 ÷ 50	0.9996	3.1	99
